# Self-Reported Dignity among People Admitted to Psychiatric Wards and Its Association with Suicidal Behaviour: Perte de dignité auto-évaluée chez les personnes admises dans des services psychiatriques et son association avec les comportements suicidaires

**DOI:** 10.1177/07067437251355644

**Published:** 2025-07-07

**Authors:** Matthew Buchok, Harvey M. Chochinov, Sarah Kowall, Shay-Lee Bolton, Renée El-Gabalawy, Jennifer M. Hensel, James M. Bolton

**Affiliations:** 1 Department of Psychiatry, Rady Faculty of Health Sciences, 8664 University of Manitoba, Winnipeg, MB, Canada; 2 CancerCare Manitoba Research Institute, Winnipeg, MB, Canada; 3 Department of Community Health Sciences, Rady Faculty of Health Sciences, 8664 University of Manitoba, Winnipeg, MB, Canada; 4 Department of Clinical Health Psychology, Rady Faculty of Health Sciences, 8664 University of Manitoba, Winnipeg, MB, Canada; 5 Department of Psychology, 8664 University of Manitoba, Winnipeg, MB, Canada; 6 Department of Anesthesiology, Perioperative and Pain Medicine, Winnipeg, MB, Canada

**Keywords:** dignity, mental illness, inpatient, suicide

## Abstract

**Objective:**

Dignity is an important construct in vulnerable persons; however, there is limited research examining dignity in patients with mental illness. Our study aims to examine self-reported dignity-related distress among psychiatric inpatients using the patient dignity inventory (PDI) and investigate the relationship between this distress and demographic and clinical variables, including suicidality.

**Methods:**

Between June 2021 and July 2022, 97 participants were recruited from two hospitals in Winnipeg, Canada. Participants were patients admitted to acute psychiatric wards, 18 years or older, and provided informed consent. Participants completed a series of standardized self-report measures including the PDI and validated measures of depression, alcohol use, and suicidal behaviour. Demographic and clinical information was also obtained from patient charts. General linear models were used to investigate the relationship between dignity-related distress and demographic and clinical variables.

**Results:**

The majority of the study sample had moderate to high depression symptomatology (57.7%), previous psychiatric hospitalizations (67.4%), and previous suicide attempts (52.6%). Dignity-related distress was not associated with gender, sexual orientation, age, marital status, or education. Higher levels of dignity-related distress were associated with mental disorder comorbidities (*P* < 0.01), greater depressive symptoms (*P* < 0.001), and higher risk alcohol use behaviours (*P* < 0.001). Increasing levels of dignity-related distress were associated with greater intensity of suicidal ideation (*P* < 0.001) having at least one previous suicide attempt (*P* < 0.001), and having a high desire to die during that attempt (*P* < 0.001).

**Conclusion:**

Among psychiatric inpatients, impairment in their sense of dignity was associated with greater clinical severity including both a history of suicide attempt and current suicidal ideation. Further investigation may lead to targeted interventions to mitigate dignity-related distress and improve patient outcomes.

## Abrégé

**Objectif:**

La dignité est une notion importante chez les personnes vulnérables; cependant, les recherches sur la perte de dignité chez les patients atteints de maladie mentale sont limitées. Notre étude vise à examiner la détresse liée à la perte de dignité auto-évaluée chez les patients hospitalisés en psychiatrie à l’aide du Portrait de la dignité du patient et à étudier la relation entre cette détresse et les variables démographiques et cliniques, y compris la suicidalité.

**Méthodes:**

Entre juin 2021 et juillet 2022, 97 participants ont été recrutés dans deux hôpitaux de Winnipeg, au Canada. Les participants étaient des patients admis dans des services de soins psychiatriques actifs âgés de 18 ans ou plus et avaient fourni un consentement éclairé. Les participants ont effectué une série de mesures normalisées auto-déclarées, y compris le Portrait de la dignité du patient et des mesures validées de la dépression, de la consommation d’alcool et des comportements suicidaires. Des renseignements démographiques et cliniques ont également été obtenus à partir des dossiers des patients. Des modèles linéaires généraux ont été utilisés pour étudier la relation entre la détresse liée à la perte de dignité et les variables démographiques et cliniques.

**Résultats:**

La majorité de l’échantillon de l’étude présentait des symptômes de dépression modérés à élevés (57,7 %), des antécédents d’hospitalisation psychiatrique (67,4 %) et des antécédents de tentatives de suicide (52,6 %). La détresse liée à la perte de dignité n’était pas associée au genre, à l’orientation sexuelle, à l’âge, à l’état matrimonial ou au niveau d’éducation. Des niveaux plus élevés de détresse liée à la perte de dignité étaient associés à des comorbidités mentales (*P* < 0,01), à des symptômes de dépression plus importants (*P* < 0,001) et à des comportements à risque liés à la consommation d’alcool (*P* < 0,001). Les niveaux croissants de détresse liée à la perte de dignité étaient associés à une intensité accrue des idées suicidaires (*P* < 0,001), à au moins une tentative de suicide antérieure (*P* < 0,001) et à un désir intense de mourir lors de cette tentative (*P* < 0,001).

**Conclusion:**

Chez les patients hospitalisés en psychiatrie, une altération du sentiment de dignité était associée à une plus grande gravité clinique, notamment à des antécédents de tentative de suicide et à des idées suicidaires actuelles. Des recherches plus poussées pourraient mener à des interventions ciblées visant à atténuer la détresse liée à la perte de dignité et à améliorer les résultats pour les patients.

## Introduction

Dignity is a complex concept that has been formulated as both an inherent self-worth, common to all people, and an acquired sense of self-worth, influenced by interactions with others in society.^1^ Dignity encompasses concepts of respect, acceptance, self-esteem, and autonomy; these concepts can be threatened by illness, and underscore the importance of medical care that is centred on compassion and an understanding of the person.^2,3^ Within medical literature there has been an increasing appreciation for the importance of dignity, particularly in the field of palliative care.^3–8^ Patients identify issues that may impinge upon or enhance their sense of dignity, and injury to a patient's sense of dignity has been clearly shown to worsen distress.^3,4,6–8^ Deficits in dignity have been found to be associated with harmful consequences for patients including feelings of fear, humiliation, embarrassment, insecurity, frustration, isolation, apathy, depression, and loss of will to live.^3,4,6,9–12^ Further, previous studies have documented that loss of dignity was the most common reason cited by patients who had opted for medical assistance in dying (MAiD), being cited in 50–60% of cases.^13,14^ Preserved dignity, on the other hand, has been found to be associated with better adherence to treatment, optimization of preventative care, and higher satisfaction with healthcare encounters.^9–12^ It has also been demonstrated that interventions targeted towards improving dignity can significantly reduce depressive symptoms, decrease suffering, and increase will to live.^5^

Compared to palliative care, dignity has been relatively underexplored among people with mental illness. It would be reasonable to expect that mental disorders could have a significant impact on a person's sense of dignity, particularly in light of the psychological factors associated with dignity shown in primarily palliative care research. Patients with mental health conditions commonly face stigma and discrimination, resulting in violations of civil, cultural, political, and social rights.^15,16^ Like palliative care, psychiatry often involves conditions that are chronic and functionally impairing, frequently with a focus on symptom management and improving quality of life. Similar to patients who are facing death, patients with mental health conditions may contemplate their illness as an assault on their former sense of self, potentially affecting their sense of identity, autonomy, and self-worth; which, in turn, could alter their perception of whether life is worth living and their feelings regarding a hastened death. Given that loss of dignity is a profound predictor of the wish to die in the terminally ill,^14^ there may be a similar association between loss of dignity and suicidality in patients with mental health conditions. These perspectives gain considerable importance, given legislative initiatives contemplating MAiD for persons whose sole underlying medical condition is mental illness.^17^

To better define the construct of dignity, Chochinov et al. developed the model of dignity in the terminally ill through analysis of the understanding and perceptions of dignity among patients with advanced stage terminal cancer.^3^ This model considers areas of illness-related concerns, dignity conserving repertoire, the social dignity inventory, and encapsulates physical, psychosocial, spiritual, and existential issues, allowing them to be consolidated into the concept of dignity-related distress.^3,4,7^ The themes and subthemes of this model were then used to develop the patient dignity inventory (PDI), a 25-item questionnaire that provides a measure of dignity-related distress and assesses a broad range of issues that influence sense of dignity.^7^ Previous studies have demonstrated the reliability and internal consistency of the PDI when applied to psychiatric populations.^11,18^ These studies found that the PDI was easily understood and applied in a psychiatric setting, and that higher scores of dignity-related distress were significantly associated with higher scores of anxiety and depression in a sample of psychiatric inpatients.^11,18^ Another study using the PDI to measure dignity-related distress in a sample of psychiatric inpatients found that increased dignity-related distress was significantly associated with higher perceived coercion on admission, better insight, and more negative symptoms.^19^ To our knowledge, no prior studies have examined the relationship between dignity and suicidality in patients with mental health conditions using empirically supported tools. The aim of this study is to explore the experience of dignity-related distress among people hospitalized in psychiatric wards, and investigate the relationships between dignity and demographic and clinical variables, with a particular focus on suicidality.

## Methods

### Setting and Participants

Our study sample was comprised of people receiving acute inpatient psychiatric care in general adult psychiatric wards within two acute care hospitals in Winnipeg, Canada. Recruitment of participants occurred between June 2021 and July 2022. Potential study subjects were identified by nursing staff and the research team based on the following inclusion criteria: 18 years or older, current inpatients during the study period, possessing the capacity to provide valid and informed written consent, and a degree of fluency in the English language allowing for completion of the assessment measures. Patients were not approached if they were currently agitated or emotionally dysregulated. Potential participants were then given verbal and written information regarding the study. After providing informed, written consent, participants completed a demographic information sheet, the PDI, the 9-item Patient Health Questionnaire (PHQ-9), the Alcohol Use Disorders Identification Test (AUDIT), and the Beck Scale for Suicide Ideation (BSS). During this time the research team obtained additional information from the patients’ medical record including mental disorder diagnoses, admission history, and current length of stay.

The University of Manitoba Health Research Ethics Board approved this study [No. HS24508 (H2020:532)], with site-specific Hospital Research Review Boards at the Health Sciences Centre and Victoria General Hospital granting approval for gathering information from patients and accessing medical records.

### Patient Demographics

Demographic information was obtained from patient charts as well as patient self-report. Chart data included age (categorized into quartiles: 18–23 years, 24–32 years, 33–47 years, 48 years and older), length of stay (in days) at the time of test administration (categorized into quartiles: 1–3 days, 4–7 days, 8–18 days, 19+ days), history of previous admissions (none, one, multiple), and primary mental disorder diagnosis (categorized into bipolar disorder, psychotic disorder, depression/anxiety, and other). The “other” group of mental disorders was largely composed of patients with personality disorders, personality traits, or eating disorders. Alcohol and drug use disorders were typically comorbid with other primary mental disorders and therefore were not included as a primary diagnosis category. Comorbidities included additional mental disorders and physical illnesses, and were grouped into any mental health diagnoses, any physical health diagnoses, presence of both, or none. Self-report data included gender identity [male (cis male or trans male), female (cis female or trans female), or gender diverse (nonbinary, two-spirit, or other with open response)], sexual orientation [heterosexual or LGBTQ2SIA+ (gay/lesbian, bisexual, asexual, or other with open response)], level of educational attainment (postsecondary or high school or less), and marital status (single, separated/divorced, or married/common-law).

### Measures

#### Patient dignity inventory

**Figure 1.**
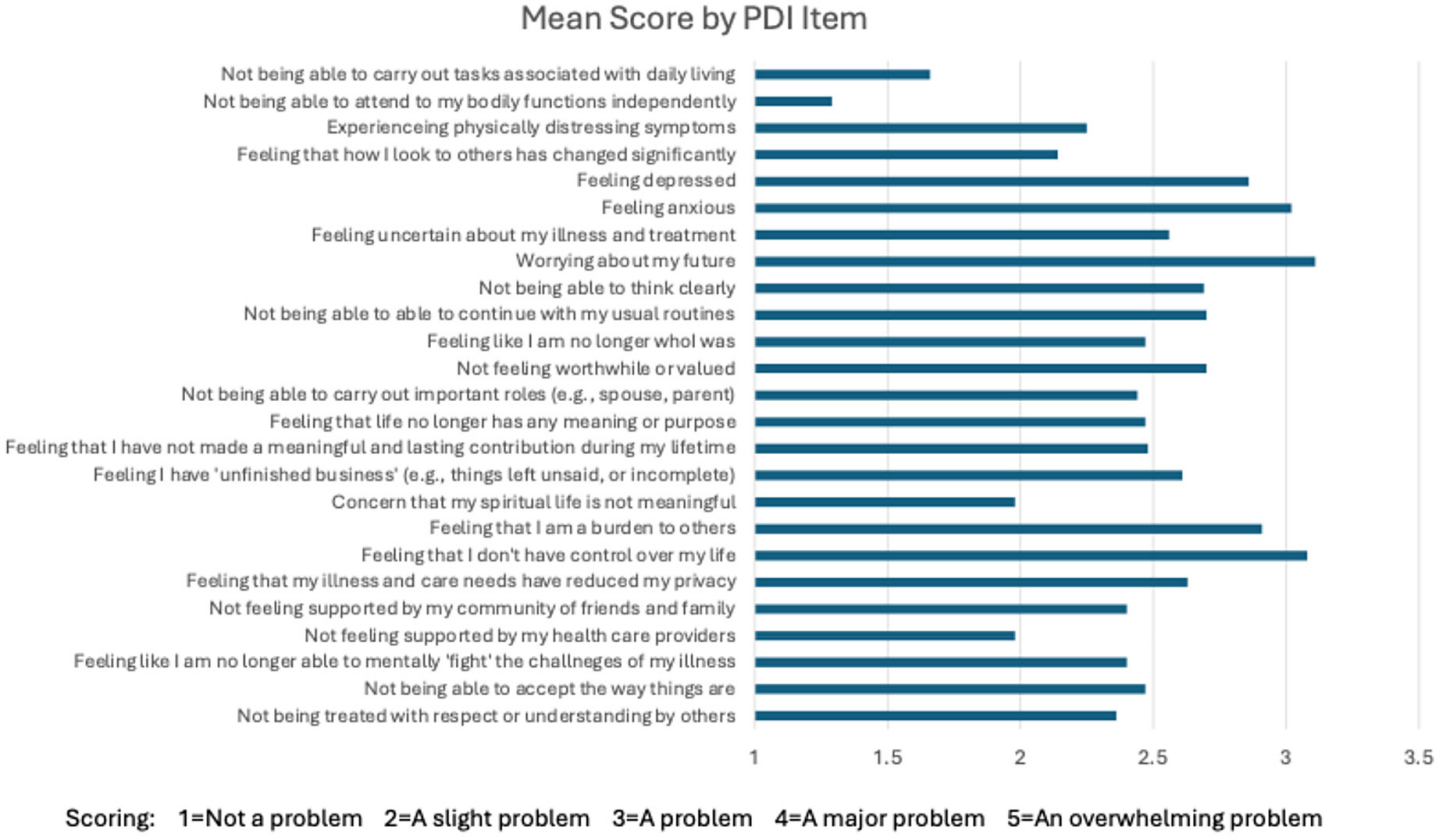
The patient dignity inventory. People completing the PDI are instructed as follows: “For each item, please indicate how much of a problem or concern these have been for you within the last few days.” Scores range from 1 to 5 as described above. The figure presents the mean score for each item. PDI = patient dignity inventory.

To assess dignity-related distress, participants completed the 25-item PDI (Figure 1).^7^ This self-report measure asks participants how much of a problem or concern each item has been for them within the last few days. Each item is rated using a 5-point scale (1 = not a problem; 2 = a slight problem; 3 = a problem; 4 = a major problem; 5 = an overwhelming problem), yielding a total score ranging from 25 to 125, with higher scores indicating a greater loss of dignity. Throughout the analysis, we treated the PDI scores as a continuous measure. The PDI shows excellent internal consistency, high test–retest reliability, and high validity (Cronbach's α = .93, *r* = .85).^7^

#### Mental health symptomatology

Depressive symptoms were assessed using the 9-item self-report PHQ-9 using a 4-point Likert scale (0 = “Not at all” to 3 = “Nearly every day”) to assess symptoms within the past 2 weeks.^20^ Scores ranged from 0 to 27 with a score of 0–4 indicating no depressive symptoms, 5–9 indicating mild depressive symptoms, 10–14 indicating moderate depressive symptoms, 15–19 indicating moderately severe depressive symptoms, and 20–27 indicating severe depressive symptoms.^20^ In our analysis, participant scores were categorized into two groups with scores of 0–9 representing those with absent or mild depressive symptoms (reference group), and scores of 10–27 representing those with moderate to severe depressive symptoms, in line with prior work suggesting clinical thresholds.^20^ The PHQ-9 has been demonstrated to be a reliable and valid measure of depressive symptom severity.^20^

Symptoms of alcohol use disorder were measured using the 10-item self-report AUDIT, a valid and reliable measure.^21^ Items in this measure examined the frequency of alcohol intake, alcohol dependence, and alcohol-related harm. The first eight items are scored on a scale from 0 to 4, while the last two items are scored as either 0, 2, or 4.^22^ Scores can range from 0 to 40, with 0 indicating no problem with alcohol. Scores of 1–7 suggest low-risk consumption, scores of 8–14 suggest hazardous or harmful alcohol consumption, and scores of 15+ suggest likely alcohol dependence or moderate–severe alcohol use disorder.^22^ In our analysis, we formed two groups, one being those with no to low-risk consumption (0–7; reference group) and the other being those with moderate to high-risk consumption (8+).

Suicidal thoughts and behaviour were assessed using the 21-item BSS.^23,24^ Participants were asked to rate the items on a 3-point scale ranging from 0 to 2, with higher scores indicating greater severity of suicidality. The first five items screen for suicidal ideation. Those who score 0 on the first five items are required to skip questions 6–19, which ask about thoughts or plans of suicide if the participant indicated they had suicidal ideation in questions 1–5. The total score of the first 19 items ranges from 0 to 38, assessing the intensity of suicidality in each participant. Item 20 on the BSS asked participants if they have ever attempted suicide. If participants indicated that they had attempted suicide in the past, they go on to answer item 21, which asks about their desire to die during the attempt.^24^ Individuals who were valid skips to any one question on the BSS were coded as a 0 “not a problem” to that item given that they did not experience it. Four variables were created based on questions from the BSS: (1) Total BSS score was calculated based on responses to questions 1–21 across all respondents, (2) dichotomous suicidality (none vs. some), (3) number of suicide attempts was assessed in question 20 (0, 1, 2, or more attempts), and (4) desire to die (low/no desire to die, moderate, or high desire to die).

### Analysis

Mean scores were calculated for the responses to each item on the PDI. Rates were determined for the demographic and clinical variables within the sample. We then assessed the relationship between demographic and clinical health correlates (independent variables) and dignity-related distress (PDI score; dependent variable) using univariate general linear models (GLMs). The association between dignity-related distress (PDI score; independent variable) and suicidality (as measured on the BSS; dependent variable) was assessed, again using GLM. Estimated marginal means, standard errors, unstandardized coefficients, degrees of freedom, *F*-values, and *P*-values were reported from the GLM. Posthoc contrasts were used to test for significant differences between variable categories among multilevel categorical variables.

## Results

Between June 2021 and July 2022, 192 patients were identified to the research team as potential participants. Of those, 88 patients reported that that they were not interested in the study. Of the 104 patients who provided consent, seven dropped out prior to completing the study, each reporting that they no longer felt like continuing. This left a final sample of 97 participants. Descriptive characteristics of the sample are presented in Table 1. Less than 5% of data was missing for any variable. The sample was predominantly heterosexual (76.9%) and single (62.2%), and 52.1% was male. The most common primary mental health condition was psychotic disorder (47.4% of the sample).

**Table 1.** Demographics and Scale Scores Among the Study Sample (*N* = 97).

Variables	*N* (%)
Gender identity (*N* = 94)	
Male	49 (52.1)
Female	38 (40.4)
Gender diverse	7 (7.4)
Sexual orientation (*N* = 91)	
Heterosexual	70 (76.9)
LGBTQ2SIA+	21 (23.1)
Age (*N* = 97)	
18–23 years	24 (24.7)
24–32 years	23 (23.7)
33–47 years	26 (26.8)
48+ years	24 (24.7)
Marital status (*N* = 90)	
Single	56 (62.2)
Separated/divorced	11 (12.2)
Married/common law	23 (25.6)
Education (*N* = 91)	
High school or less	47 (51.6)
Postsecondary	44 (48.4)
Length of stay (*N* = 95)	
1–3 days	21 (22.1)
4–7 days	23 (24.2)
8–18 days	28 (29.5)
19+ days	23 (24.2)
Primary diagnosis (*N* = 97)	
Bipolar disorder	19 (19.6)
Psychotic disorder	46 (47.4)
Depression/anxiety	19 (19.6)
Other	13 (13.4)
Comorbidity (*N* = 97)	
None	30 (30.9)
Mental health diagnosis	29 (29.9)
Physical health diagnosis	24 (24.7)
Both	14 (14.4)
Previous hospitalizations (*N* = 95)	
None	31 (32.6)
One	20 (21.1)
Multiple	44 (46.3)
PHQ-9 (*N* = 97)	
None/mild (0–9)	41 (42.3)
Moderate/severe (10–27)	56 (57.7)
AUDIT (*N* = 97)	
Low risk (0–7)	68 (70.1)
Moderate/high risk (8+)	29 (29.9)
Any suicidality	
No suicidality	37 (38.1)
Some suicidality	60 (61.9)
Suicide attempts	
No suicide attempts	46 (47.4)
One suicide attempt	19 (19.6)
Two or more suicide attempts	32 (33.0)
Desire to die during suicide attempt	
Low/no desire to die	18 (18.6)
Moderate desire to die	10 (10.3)
High desire to die	69 (71.1)

*Note*. PHQ-9 = 9-item Patient Health Questionnaire; AUDIT = alcohol use disorders identification test.

Figure 1 presents the 25 items assessed in the PDI, along with the mean scores for each item. Most items had a mean score between 2 and 3. The four items with a mean score below 2 (i.e., scoring between “not a problem” to “a slight problem”) were Item 1 (Not being able to carry out tasks associated with daily living), Item 2 (Not being able to attend to my bodily functions independently), Item 17 (Concern that my spiritual life is not meaningful), and Item 22 (Not feeling supported by my healthcare providers). There were three items with a mean score over 3 (“a problem”): Item 6 (Feeling anxious), Item 8 (Worrying about my future), and Item 19 (Feeling like I don’t have control over my life). The mean score for the total PDI scale was 61.7.

Table 2 shows the association between sample characteristics and dignity-related distress. Comorbidity demonstrated a significant relationship with dignity-related distress [*F*(3, 93) = 4.159, *P* = 0.003]. Posthoc analysis revealed that individuals with comorbid mental disorders had a higher level of dignity-related distress than those without a comorbid condition (*P* = 0.012). People with moderate to severe depression had significantly greater dignity-related distress (mean = 74.73, SE = 2.42) when compared to those with no or low depressive symptoms [mean = 43.73, SE = 2.83; *F*(1, 95) = 69.301, *P* < 0.001]. Moderate- and high-risk alcohol use was also associated with greater levels of dignity-related distress [*F*(1, 95) = 10.043, *P* = 0.002].

**Table 2.** General Linear Models Exploring the Association Between Demographic and Clinical Health Correlates and Total PDI Score.

Independent variable	Mean PDI score	SE	Unstandardized coefficient		*df*	*F*	*P*
			*B*	SE			
Gender identity					2,91	2.844	0.063
Male	56.90	3.35	Ref.				
Female	68.16	3.80	11.26	5.07			
Gender diverse	53.90	8.86	−3.04	9.47			
Sexual orientation					1,89	2.479	0.119
Heterosexual	59.61	2.83	−9.29	5.90			
LGBTQ2SIA+	68.90	5.18	Ref.				
Age					3,93	2.045	0.113
18–23 years	60.46	4.76	4.50	4.76			
24–32 years	58.22	4.86	2.26	6.73			
33–47 years	70.96	4.57	15.03	6.60			
48+ years	55.96	4.76	Ref.				
Marital status					2,87	.446	0.642
Married/common law	58.87	3.19	Ref.				
Single	64.11	7.19	5.24	5.91			
Separated/divorced	60.09	4.97	1.22	8.75			
Education					1,89	0.008	0.927
High school or less	62.04	3.42	−0.45	4.91			
Postsecondary or higher	61.59	3.53	Ref.				
Length of stay					3,91	0.971	0.410
1–3 days	63.19	5.21	−4.16	7.21			
4–7 days	62.35	4.98	−5.00	7.04			
8–18 days	56.11	4.51	−11.24	6.72			
19+ days	67.35	4.98	Ref.				
Primary diagnosis					3,93	2.521	0.063
Bipolar disorder	56.16	5.31	−17.00	8.34			
Schizoaffective/psychotic	57.61	3.41	−15.55	7.27			
Depressive/anxiety	68.95	5.31	−4.21	8.34			
Other	73.15	6.42	Ref.				
Comorbidity					3,93	4.159	0.008
None	53.17	4.13	Ref.				
Mental health diagnosis	71.72^a^	4.20	18.56	5.89			
Physical health diagnosis	56.25	4.62	3.08	6.19			
Both	68.07	6.04	14.91	7.32			
Previous hospitalizations					2,92	2.629	0.078
None	54.10	4.22	Ref.				
One	67.45	5.25	13.35	6.73			
Multiple	64.89	3.54	10.79	5.50			
Depression symptoms (PHQ-9)					1,95	69.301	<0.001
None/mild (0–9)	43.73	2.83	Ref.				
Moderate/severe (10–27)	74.73	2.42	31.00	3.72			
Alcohol use (AUDIT)					1,95	10.043	0.002
Low risk (0–7)	56.87	2.75	Ref.				
Moderate/high risk (8+)	72.79	4.21	15.93	5.03			

*Note*. PDI = patient dignity inventory; PHQ-9 = 9-item Patient Health Questionnaire; AUDIT = alcohol use disorders identification test.

^a^ Significantly different from the reference group (*P* = 0.012).

The relationship between suicidality and dignity is presented in Table 3. Higher levels of dignity-related distress was significantly associated with greater intensity of suicidality, as measured by the BSS total score [*F*(1, 95) = 55.704, *P* < 0.001] and any endorsement of prior suicidality [*F*(1, 95) = 50.441, *P* < 0.001]. Higher dignity-related distress scores were significantly linked with a history of one (mean = 69.42, SE = 4.82) or several (mean = 73.94, SE = 3.72) suicide attempts, compared to no history of attempt [*F*(2, 94) = 14.013, *P* < 0.001]. Higher dignity-related distress scores were also significantly related to a desire to die [*F*(2, 94) = 15.395, *P* < 0.001]. Posthoc analysis revealed that higher dignity-related distress was associated with a moderate desire to die during a prior attempt (mean PDI = 70.90, SE = 6.57) and a strong desire to die during a prior attempt (mean PDI = 83.89, SE = 4.90), compared to a low or no desire to die (mean PDI = 54.48, SE = 2.50).

**Table 3.** General Linear Models Exploring the Association Between PDI Scores and Suicide Correlates from the Beck Scale for Suicide Ideation (BSS).

Independent variable			Unstandardized coefficient				
	Mean PDI score	SE	*B*	SE	*df*	*F*	*P*
BSS total score	—	—	1.37	0.18	1,95	55.70	<0.001
Any suicidality					1,95	50.441	<0.001
No suicidality	43.95	3.17	Ref.				
Some suicidality	72.53	2.49	28.59	4.03			
Suicide attempts							
No suicide attempts	49.85	3.10	Ref.		2,94	14.013	<0.001
One suicide attempt	69.42^a^	4.82	19.57	5.73			
Two or more suicide attempts	73.94^a^	3.72	24.09	4.84			
Desire to die during suicide attempt					2,94	15.395	<0.001
Low/no desire to die	54.48	2.50	Ref.				
Moderate desire to die	70.90^b^	6.57	16.42	7.03			
High desire to die	83.89^a^	4.90	29.41	5.50			

*Note*. PDI = patient dignity inventory.

^a^ Significantly different from the reference group (*P* < 0.001).

^b^ Significantly different from the reference group (*P* = 0.022).

## Discussion

This study examined the relationship between dignity, demographic, and clinical characteristics, and suicidal behaviour in a sample of acute psychiatric inpatients. To our knowledge, this is the first study to demonstrate a significant association between patient reported dignity and suicidality in patients with mental health conditions. Suicidal ideation, a history of suicide attempts, and a strong desire to die when attempting suicide were all factors that correlated with impairments in a person's sense of dignity. With dignity not being a typical point of focus in psychiatric assessments, despite its established connection with desire for death among palliative care patients, a deeper understanding of its psychological impact in people suffering from mental disorders is warranted.

While there are no prior studies that have investigated the relationship between dignity and suicidality in psychiatric inpatients, these findings are supported by previous studies in palliative care, which found that loss of dignity was associated with loss of will to live and was the most common reason cited by patients who requested a hastened death.^12–14^ This relationship is not unexpected. Much like palliative care patients who are facing death, patients with mental health conditions may be confronted with concerns regarding their identity, autonomy, and generativity. This raises the question of whether therapies aimed to preserve or increase dignity, which have been demonstrated to increase will to live in palliative care patients,^5^ may reduce suicidality in patients with mental health conditions. Given policy considerations that would see MAiD being available for people whose sole underlying condition is serious mental illness, future research should not only clarify the association between dignity, suicidality, and MAiD requests, but also whether dignity enhancing interventions reduce the desire for MAiD among people with mental illness.

This study also provides novel findings regarding the significant relationship between dignity and specific clinical facets of mental illness, namely depression and problematic alcohol use. Patients who were found to have moderate or severe depressive symptoms on the PHQ-9 reported significantly elevated dignity-related distress compared to patients with mild or no depressive symptoms. This finding is consistent with previous studies in psychiatric inpatients, which showed a correlation between PDI score and depression.^11,17^ Interestingly, interventions that improve dignity in palliative care patients significantly reduced depressive symptoms.^4^ The use of similar therapies in mental health inpatients may prove helpful in mitigating symptoms of depression. Patients with scores indicating moderate to high-risk alcohol use were found to have significantly worsened dignity-related distress compared to patients in low risk or no use groups. This finding supports previous studies that have outlined the significant impact of stigma and discrimination on patients who experience substance use problems, and the potential for these patients to experience violations of their human rights and dignity.^25^ This relationship is also of interest given that alcohol use as a means of coping with distress is a known phenomenon that has been found to lead to higher risk drinking practices and associated with initiation, maintenance, and relapse of substance use.^26–29^ Reduction in dignity-associated distress may therefore reduce drinking as a coping mechanism and decrease high risk patterns of alcohol use. This idea is supported by a previously published case study in which a patient with severe alcohol use disorder attained abstinence through participation in dignity therapy, after having failed other outpatient treatment modalities.^30^

There are several limitations that should be considered. Our sample size of 97 patients is relatively small and may introduce a potential source of error. With 95 of the approached patients refusing to be involved in the study, it is likely that selection bias influenced the results. It is conceivable that nonparticipants had either higher or lower levels of both dignity distress and suicidal behaviour, which could artificially inflate results or bias them towards the null. Future studies should address this bias by examining the profile of nonparticipants, including reasons for nonparticipation, if possible. Additionally, our sample included patients with a wide variety of mental disorders and reasons for admission. By analysing this heterogenic population together, it is possible that certain subgroups of diagnoses with stronger relationships with dignity-related distress may have been missed. Future research regarding dignity-related distress and its relationship with patient factors and symptomatology in specific disorders would be a valuable addition to the literature. An example would be borderline personality disorder, with clinical features that may be anticipated to correlate with dignity-related distress including chronic feelings of emptiness, unstable sense of self, and recurrent suicidal behaviour. Although there was no upper age limit in the inclusion criteria, this study sampled from general adult wards, and did not sample from the psychiatric ward in Winnipeg dedicated to people over age 65. A future study that examines older psychiatric inpatients would provide important information on dignity-related distress in this population. Another limitation is that the study was cross-sectional in design, and therefore we can only report associations, and not causality, regarding dignity and suicidality. This study did not examine income or housing status, which are demographic factors associated with both mental illness and suicidal behaviour. Furthermore, the study was conducted during the COVID-19 pandemic, which exacerbated the relationship between lower household income level and suicidal behaviour.^31^ Finally, the study sample consisted entirely of hospitalized patients and therefore it is unknown if the results are generalizable to other clinical groups, or people with mental disorders who have not sought treatment. Further research in other settings such as outpatient clinics would be helpful in improving our understanding.

Our study is the first to demonstrate a relationship between dignity-related distress and suicidality among psychiatric inpatients on general acute care wards. These findings carry potentially significant clinical implications regarding the management of inpatients on psychiatric wards, both in terms of the assessment of dignity distress as well as therapeutic approaches that directly aim to enhance dignity. Further research in this area is of paramount importance to improve the lives of people with mental illness, who appear to suffer from considerable dignity-related distress.
